# Professional Ethics

**Published:** 1873-12

**Authors:** Wm. F. Thompson


### "PROFESSIONAL ETHICS."

BY WM. F. THOMPSON.

The following thoughts occur, in answer to an article I have just finished reading. I agree with your contributor ;—in Aug. No. of the REGISTER,—high-sounding titles are but empty things, unless backed by the merit that justifies their possession and *should only be conferred* as a "reward of merit." He who gathers them for speculative purposes dishonors not only himself, but that which should be a *part of himself*,—his profession—thereby losing the respect and esteem which are justly due a conscientious thinker and worker, and the confidence of his patrons, overwhelming him ultimately in *pecuniary loss* and *professional disgrace* ; but when *worthily bestowed*, in consideration of sterling traits that make the *standard social* and *professional man*, it is a *badge of honor*, and should be worn proudly, yet sacredly and in fear, lest some *unworthy act might mar its beauty*, paling its splendor, *when it should shine*

*an undimmed light*,—an "Eastern Star"—as an incentive to those who have not reached that *standard of acknowledged excellence.*

While I agree with your contributor in the main, and accept a part of what he says as good, wholesome advice to myself, I most emphatically disagree with him upon one point; for, unless rightly understood, it will undoubtedly mislead some who are looking for landmarks to guide them in their course. There is no danger of a student's too thoroughly educating himself before commencing the *great life-work* that is before him. Unfortunately, too many turn themselves out, *half made up*, working at a disadvantage ever after; for it is difficult to unlearn, and learn again what should have been sought for in the right spirit at first. The objectionable paragraph is this; viz.,

"The Drs., M. D. and D. D. S. upon cards and signs are but little guarantee of ability. Society has so often been cheated by them, that he who flourishes those titles most, ever making them conspicuous, is looked upon with distrust. They are no mark of brains; their profession, or rather the knowledge which their possessor is supposed to have, is highly important. They should only be regarded as an evidence that the object had been sought for—not as the end sought."

What a fearful commentary upon our medical and dental schools! If they are no "guarantee of ability," what have we for protection? and where does this trouble lie? for there must be some lacking thing, upon the part of teacher or pupil, to produce so disastrous a result as pictured. I admit, "society" has been often "cheated;" but should that disgrace a passport, when honorably obtained and professionally regarded?

Because a few unprincipled ones have been recreant to a *sacred trust*, does that in any way reflect upon him or them who have striven for, and with much sacrifice possessed themselves of, the coveted prize? Should we forego the honor because it makes us "conspicuous" over those who have them not?

"They are no mark of brains." Why? If not, it reflects severely upon those who sit in judgment in high places, with

*vested power* to award or withhold. Mistakes are made, no doubt, but when a student manifests no pride or interest in preparing himself for a vocation that is to be his life calling, every impediment should be thrown in his way to dissuade him from proceeding farther with what will surely prove a mistaken step; while in some other pursuit, more congenial to his tastes, he might rise to an enviable position. Instead of living out his allotted time, grinding at a wheel that must inevitably prove a treadmill, let him be occupied in something, that—because he loves it—will prove a success.

All have not been able to attend a regularly chartered institution of learning; but if they are *capable* and *worthy*, this need not debar them from obtaining justice nor will justice be denied when properly petitioned for. To those commencing,—and to them I more particularly allude,—it is especially desirable that they attend some good school, thereby avoiding many vexed questions that otherwise must occur, by a self—only—examination. This chart and compass has been provided, that none may drift about, tempest-tossed, discourdged and ultimately disgusted—not unfrequently to the disgust of others. No words can I find in the English vocabulary, sufficiently strong to express my unqualified condemnation and utter reprehension of those who, having opportunities, fail to improve them. The world places a high estimate upon a well-stored mind, enriched by culture and sparkling with the ripe fruit of sagacity and golden good sense, which makes the possessor "conspicuous" among many. These become a mighty lever, and lift us above the reproach that has been too frequently cast upon us. We observe and others estimate our calling, in different localities, by the standard of the men representing it.

Again: it is alleged that degrees "should only be regarded as an evidence that the object—i. e. an education—had been sought for." This to me is a most surprising assumption, which cannot be the offspring of intelligent reflection, since it is in direct conflict with the settled judgment of every country and of every age. When the highest learned and legally constituted body in a state confers a mark of distinguished exeellence upon a man, the presumption is, that it ha been worthily and well bestowed. Unless the honesty of the body be impeached, every man is bound to believe that the party possessing it has successfully established his claim to it, and therefore he is entitled to the confidence of the community; while the man without such established authority must prove to mankind *what he is*, before he can claim equal status. The foundacion of the former is laid: that of the latter has no existence. The difference is as wide asunder as the poles.

Because we have eminent self-educated men in our profession,—men who *will have no barrier* 'twixt them and success; who persistently sought, with a never-tiring earnestness, that which they felt was indispensably necessary to fit them for the position in life they should occupy,—it is no argument that *we* should deny ourselves a shorter and an easier method of gaining the same information.

It matters not, if a man *is qualified*, from what source he became possessed of those qualifications; but it is better for the student—and for the profession at large—that he makes his *first blunders* inside the college walls. This one thing, on the part of would-be-dentists-before-their-time, has done more to destroy confidence, hold back and cripple us, than we can estimate. Until the student fully comprehends—and I know of but one way to accomplish this result; viz., legislation—that he is not permitted to manipulate his unskillful hands upon a *too ever ready* and confiding public, charging an unprofessional fee, because *not yet* professional, and fully knowing this, just so long must we suffer and wait, the misdeeds of a few reflecting upon the many. Until the same demands are made, and rigidly enforced, as with our sister profession,—medicine,—we cannot hope to have them fraternize with us. To remedy this evil, let us stamp in flaming characters upon our banner, "Excelsior," and by our daily actions prove our interest in the work of reformation.

